# The Importance of Health Information on the Internet: How It Saved My Life and How it Can Save Yours

**DOI:** 10.2196/16690

**Published:** 2019-10-27

**Authors:** Andre Kushniruk

**Affiliations:** 1 School of Health Information Science University of Victoria Victoria, BC Canada

**Keywords:** patient journey, human factors, consumer health informatics, eHealth, digital health, participatory medicine, shared decision-making, cancer information, tongue cancer

## Abstract

The internet holds the potential promise of improved patient outcomes, especially when one is faced with a critical or life-threatening disease or condition. Appropriate and timely access to health information can support informed negotiation of optimal treatments, optimal management, and expedited recovery, and to an improved outcome for a patient. However, there are many human and technical barriers that may prevent the application of the best possible information for both patient and provider alike, making the patient journey complex and potentially dangerous. In this viewpoint paper, the author (who is also a JMIR editor) reflects on a personal patient journey, where use of the internet facilitated a means of reaching a good patient outcome in the face of a variety of informational and organizational limitations and gaps. This journey illustrates the importance of human-related factors affecting access to health information. The application of a range of internet information resources at critical points can result in a positive patient outcome, as this case illustrates. This paper reflects on how the experience highlights several information needs and concerns. It also highlights the need for improved access to appropriate health information along the patient journey that can support patient and provider joint decision-making. This access to information can make the difference between positive clinical outcomes and death, illustrating how health information on the internet can be both critical and life saving.

## Introduction

The Journal of Medical Internet Research (JMIR) is now celebrating its 20th anniversary and has become a leading journal in the area of health informatics and digital health, particularly in the area of using the internet to provide health information to health care providers, patients, and average citizens alike. The importance of the focus of the journal (and its sister JMIR journals) cannot be overstated, which I especially recognize due to a recent health experience that I would like to relate in this viewpoint paper. I believe it has deep meaning for me, but I also believe that it has implications for the health care system and patient interactions with it. My case will hopefully highlight some of the issues related to availability of information for patients, access to the best possible information, and how that information needs to be used and acted on to lead to the best possible patient health outcomes. In this story, the central theme is the need for access to relevant health information on the internet and translating that information into the best possible care.

## My Patient Journey

I am the editor-in-chief of JMIR Human Factors, a health informatics specialist (working both in academia and in industry in Canada and worldwide) and have been working in the field for over 25 years, but had never been on the other side of the health care system as a patient. This all changed dramatically for me in the fall of 2017 when I was diagnosed with advanced stage tongue cancer. The cancer had been missed in the preceding summer by two dentists (my condition was attributed to other, more minor problems, as it was judged unlikely to be cancer because I had none of the risk factors). By the time I reached my general practitioner, I had realized there was something more serious behind my symptoms and I entered the waiting phase (approximately a month at that point) to see an ear, nose, and throat (ENT) specialist on what was supposed to be an urgent visit. While waiting for this appointment I began using the internet to explore the possible causes of my problem, as my symptoms worsened rapidly. After waiting several weeks to see the specialist in my local area, the problem had become exacerbated. At that time, I was told I had a tumor and would require a biopsy and imaging results before I could proceed to treatment (which took several additional weeks in total to get). This was shocking news for me, but it was nowhere near as shocking as when a few weeks later the same ENT surgeon told me that the tumor was too large to operate on. As a result, I would not be receiving life-saving surgery and as a consequence I was now considered to be palliative, with a very slim statistical chance of surviving past two years.

Fortunately, my wife (an accomplished health informatician and also editor-in-chief of JMIR Nursing) decided to apply her knowledge and skill in searching the internet to examine the assessment of the specialist. Using her skills in searching PubMed, Google, YouTube, and patient blogs on the internet, she was able to determine that the situation was not completely irreversible; in other localities life-saving surgery was possible and had been conducted for patient cases very similar to mine (and reported on in the literature of peer reviewed articles). Indeed, a search of the American and Canadian cancer society websites also indicated that there was hope and that my survival chances should have been at least double what I was initially told. Through careful search of the leading articles on PubMed, we were able to locate a hospital where I would have the best chances of being cured. Coincidentally, the hospital was in New York, was a major international center, and turned out to be the part of the same organization where I had worked as an adjunct professor as far back as 2001. At that point I contacted my colleagues in New York, and within three days I was able to get an appointment with one of the top surgeons in the world and I was able to have life-saving surgery only a week later. This surgery consisted of a glossectomy and neck dissection, followed by a tongue reconstruction using leg muscle. From my first visit we discussed treatment options and I felt very comfortable, especially as all that I was told by the specialist in New York was consistent with the latest evidence available over the internet and on Medline (his group had actually generated much of the seminal research publications in the area). I was able to receive the completely successful surgery, but I did require months of recovery, follow-up radiation and chemotherapy, which I received once back in Canada.

When I returned to Canada, the focus of my attention shifted from a curative focus to how to recover from extensive surgery, chemotherapy, and radiation to regain my ability to eat and speak. Here my wife and I accessed further articles from PubMed, explored blogs and postings on YouTube and Pinterest, and posts by patients who had undergone similar treatment and therapy. This hugely helped in not only giving me realistic expectations regarding recovery (which I could not fully get from health professionals alone) but also in sharing strategies and tips for the road to full recovery. Once again, the internet became a big part of my patient journey.

I am now two years cancer free and resumed all the activities I undertook before becoming ill. Much of what happened, and how I was able to turn around what appeared to be a bleak situation, I can attribute to my friends and colleagues who helped me get the relevant information from the internet that helped with decision making about my health and well-being.

## Implications

Over the past two years I have reflected on what I have learned from this experience (as this is something I have been trained to do). My experience pointed to a variety of information gaps, issues from my human perspective, and the need for accessible, credible, and evidence-based information. Putting a human factors lens to my story, I have tried to extrapolate several lessons from my patient journey and from that of other patients I encountered during this period:

- A need for accessible information for patients on the internet (accessing this information was life saving for me) [1].
- A need for knowledge about how to access the right information. This is the advantage I had of being a specialist, but it should be made more accessible through improved user interfaces for those who do not have a background in health care [2].
- A need for access to the best possible care and treatment plans, and the ability to identify the most appropriate and best physicians available. Here the internet was what led me to locate a surgeon capable of turning my situation around [3].
- Improved electronic health (eHealth) literacy, to help in integrating technological skills with patient reasoning about critical health conditions [4,5].
- A need for credible, up to date, and substantiated evidence-based information from anywhere in the world [6].
- A need for new systems and technologies to speed up wait time and diagnosis, and to obtain second opinions (eg, easily accessible virtual second opinion systems) [7].
- A need for patients to be more informed about choices and statistics, including the meaning of survival curves in relation to different treatment options [2].
- A need for patients to be able to critique different treatment options and be provided independent advocacy and support in doing so [2].
- A need for patient education about how to select reliable and reputable information sources, requiring that information from YouTube and other such sources be curated or vetted to be up to date and useful for patient decision making [8].
- A need for integration of information and expertise, whether physical or technological (eg, a virtual tumor discussion board would have been helpful in my case) [9].
- A need for information about, and access to, life-saving treatment methods that may not be available in a patient’s local area [7].
- A need for patients to continue to provide support and advice to other patients over the internet using social media and virtual communities [3].

In conclusion, I feel my story and patient journey highlight the need for usable and useful interfaces that allow the public to access the right health information, resources, specialists, and treatment in a timely manner. Reflecting on my experience, the role of eHealth and consumer informatics for ensuring evidence-based patient choice cannot be overstated. Indeed, my patient journey highlights the need for evidence-based patient choice, which has the requirements that:

objective, unbiased information must be made available to the patient, and the patient must have the power and opportunity to choose [10].

However, the information used to support shared decision-making between the patient and provider must be of high quality. Only with timely access to the most appropriate information, along with the opportunity to select an appropriate decision path based on that information, can patients become truly empowered [11]. In line with the effort to improve the quality, access, and use of health information, I am proud and honored to be involved as an editor with JMIR, the leading journal for promoting the dissemination of scientific and evidence-based studies, methods, and approaches to improving health for patients like myself. And yes, health information on the internet can literally save your life, a message that has really resonated with me over the past two years.

## Abbreviations

eHealth: electronic health
ENT: ears, nose, and throat
JMIR: Journal of Medical Internet Research
